# Targeting Cellular Stress Mechanisms and Metabolic Homeostasis by Chinese Herbal Drugs for Neuroprotection

**DOI:** 10.3390/molecules23020259

**Published:** 2018-01-28

**Authors:** Hsiao-Chien Ting, Chia-Yu Chang, Kang-Yun Lu, Hong-Meng Chuang, Sheng-Feng Tsai, Mao-Hsuan Huang, Ching-Ann Liu, Shinn-Zong Lin, Horng-Jyh Harn

**Affiliations:** 1 Bio-innovation Center, Buddhist Tzu Chi Medical Foundation, Hualien 970, Taiwan; sharkzoe@yahoo.com.tw (H.-C.T.); scata0726@hotmail.com (C.-Y.C.); ellenellen1780@hotmail.com (K.-Y.L.); kavin273@gmail.com (H.-M.C.); spleo0825@gmail.com (M.-H.H.); sagianne@gmail.com (C.-A.L.); 2 Department of Medical Research, Buddhist Tzu Chi General Hospital, Hualien 970, Taiwan; 3 Graduate Institute of Basic Medical Science, China Medical University, Taichung 404, Taiwan; 4 Agricultural Biotechnology Center, Department of Life Sciences, National Chung Hsing University, Taichung 402, Taiwan; 5 Department of Life Sciences, National Chung Hsing University, Taichung 402, Taiwan; mrright412@gmail.com; 6 Department of Neurosurgery, Buddhist Tzu Chi General Hospital, Hualien 970, Taiwan; 7 Department of Pathology, Buddhist Tzu Chi General Hospital and Tzu Chi University, Hualien 970, Taiwan

**Keywords:** traditional Chinese medicine, herbal compounds, neuroprotection agent, neurodegenerative disease, cytokine regulation, autophagy, ER stress, glucose metabolism, synaptic function

## Abstract

Traditional Chinese medicine has been practiced for centuries in East Asia. Herbs are used to maintain health and cure disease. Certain Chinese herbs are known to protect and improve the brain, memory, and nervous system. To apply ancient knowledge to modern science, some major natural therapeutic compounds in herbs were extracted and evaluated in recent decades. Emerging studies have shown that herbal compounds have neuroprotective effects or can ameliorate neurodegenerative diseases. To understand the mechanisms of herbal compounds that protect against neurodegenerative diseases, we summarize studies that discovered neuroprotection by herbal compounds and compound-related mechanisms in neurodegenerative disease models. Those compounds discussed herein show neuroprotection through different mechanisms, such as cytokine regulation, autophagy, endoplasmic reticulum (ER) stress, glucose metabolism, and synaptic function. The interleukin (IL)-1β and tumor necrosis factor (TNF)-α signaling pathways are inhibited by some compounds, thus attenuating the inflammatory response and protecting neurons from cell death. As to autophagy regulation, herbal compounds show opposite regulatory effects in different neurodegenerative models. Herbal compounds that inhibit ER stress prevent neuronal death in neurodegenerative diseases. Moreover, there are compounds that protect against neuronal death by affecting glucose metabolism and synaptic function. Since the progression of neurodegenerative diseases is complicated, and compound-related mechanisms for neuroprotection differ, therapeutic strategies may need to involve multiple compounds and consider the type and stage of neurodegenerative diseases.

## 1. Introduction

Neurodegenerative diseases result in the progressive degeneration or death of neurons, which cannot regenerate. The loss of neurons can cause behavioral or cognitive deficits, or both. Neurodegenerative diseases, including Alzheimer’s disease (AD), Parkinson’s disease (PD), amyotrophic lateral sclerosis (ALS), and spinocerebellar atrophy (SCA), are closely related to protein aggregations [1]. There are two major types of protein aggregates in AD: the β-amyloid (Aβ) peptide derived from the amyloid precursor protein (APP), and tau protein aggregates, both of which can lead to death of neurons [2]. PD is characterized by misfolded protein aggregates. The abnormal α-synuclein protein that forms Lewy body aggregates is broadly distributed in brain regions [3,4]. ALS is caused by motor neuron degeneration in the spinal cord and cortex. Some evidence has shown that ubiquitinated proteins can accumulate in motor neurons leading to cell death [5]. SCA is a kind of polyglutamine (polyQ) disorder disease, and polyQ aggregates can cause transcriptional dysregulation leading to cellular toxicity [6].

Traditional Chinese medicine (TCM) has been practiced for centuries in East Asia. Herbs and herbal preparations are used to maintain health and cure disease. In the past few years, Chinese herbal medicines have been studied in different fields. Some Chinese herbs are known to protect and improve the brain, memory, and nervous system. However, the ingredients in various herbs are complex, and the active components in herbs must be identified to apply ancient knowledge to modern science. The extraction and analysis of components in herbs have increased in recent years [7], and many major natural therapeutic compounds in herbs have been discovered, extracted, and analyzed in recent decades [8]. Several Chinese herbs have been used to attenuate the progression of neurodegenerative diseases, and some of their active components have been identified [9,10]. After discovering an active herbal compound, the actual mechanism of the active components must be investigated.

To understand how herbal compounds attenuate neurodegenerative diseases, we summarized studies that investigated the role of herbal compounds in various neurodegenerative disease models. We categorized the compound-related mechanisms into various categories, including regulation of cytokines, autophagy, endoplasmic reticulum (ER) stress, glucose metabolism, and synaptic function.

## 2. Chinese Herbal Drugs for Neuroprotection via Cytokine Regulation

### 2.1. Neuroinflammation in the Nervous System

Neuroinflammation is usually induced by cytokines and can lead to neuronal death. In the central nervous system (CNS), microglial cells are the source of inflammatory cytokines. Proinflammatory cytokines, including interleukins (ILs), transforming growth factor (TGF)-β1, and tumor necrosis factor (TNF)-α usually make neurodegenerative disease worse. IL-1β induce neuronal injury not only by producing reactive oxygen species (ROS), but also by activating the microglial cell. It can also induce increased glycogen consumption in astrocytes, leading to elevated levels of toxic substances, thereby affecting cellular metabolism [11,12]. In addition, IL-1β and TNF-α induce neurotoxicity through glutamate production [13].

### 2.2. Cytokines and Neuronal Diseases

Neurodegenerative diseases, including AD and PD, are associated with chronic inflammatory responses [14]. Analysis of cerebrospinal fluid from patients with AD suggested that the IL-12 and IL-23 signaling pathways are activated [15]. A recent study showed that the gut microbiota regulates motor deficits and neuroinflammation in a PD model, and expressions of two proinflammatory cytokines increased in the presence of host microbiota [16]. A meta-analysis also showed higher peripheral levels of ILs in PD patients [17]. In animal study, NLRP3 (an inflammasome sensor)-deficient APP/PS1 AD mice showed improved Aβ clearance by microglia, lower Aβ-induced IL-1β formation in the brain, and decreased the Aβ-induced suppression of synaptic plasticity [18]. Furthermore, ILs can modulate the size of ischemic damage in a rodent stroke model as also found in neurodegenerative diseases [19]. IL-1, IL-6, TNF-α, and C-reactive protein (CRP) are potential targets of stroke therapy. Studies have reported that these cytokines are associated with depression [20,21,22]. According to these evidences, regulating inflammatory cytokines could be a strategy to treat neurodegenerative diseases, stroke, and mental illness. Therefore, we list some herbs that attenuate the progression of neurological disease progression through cytokine regulation.

### 2.3. Chinese Herbaldrugs that Benefit Neuronal Diseases via Cytokine Regulation

Isobavachalcone and paeonol are the main components of the herbs *Psoralea corylifolia* and *Cortex Moutan,* which were applied for PD therapy*.* In a 1-methyl-4-phenyl-1,2,3,6-tetrahydropyridine (MPTP)-induced model, both isobavachalcone and paeonol inhibited the overactivation of microglia, and decreased expressions of ILs and also prolonged the residence time of mice on a Rota-rod and alleviated neuronal necrosis [23,24].

Icariin was an anti-inflammation compound extracted from Chinese herb *Epimedium segittatum* and worked on several neuronal diseases involved in inflammatory response. In an AD animal model, icariin significantly attenuated Aβ deposition, microglial activation, and TGF-β1 immunoreactivity at amyloid plaques and restored impaired nesting ability [25]. Icariside (ICS) II, a novel phosphodiesterase 5 inhibitor derived from the herb *Epimedium brevicornum,* and tetrandrine, a bisbenzylisoquinoline alkaloid isolated from the herbal *radix Stephania tetrandra*, both inhibit inflammatory factors and improve cognitive deficits in AD animal models [26,27]. ICS II decreased levels of Aβ1-40 and Aβ1-42 and inhibited inflammatory factors, including IL-1β, TNF-α, cyclooxygenase (COX)-2, and TGF-β1, in AD. Tetrandrine treatment inhibit nuclear factor kappaB (NF-κB) activity and downregulate IL-1β and TNF-α expression.

Stroke is highly correlated with post infarction inflammation. For anti-inflammation, icariin was not only applied to AD but also stroke therapy. In an animal stroke model, icariin pretreatment reduced cytokine levels (IL-1β and TGF-β1), decreased a neurological deficit score, and reduced the infarct volume [28]. Ampelopsin (AMP), extracted from *Ampelopsis grossedentata*, attenuated neurological deficits and reduced infarct volumes, brain edema, immunoglobulin G (IgG) exudation, and neuron degeneration by inhibition of the middle cerebral arterial occlusion-induced IL-1β and TNF-α release in animal model [29]. Eriodictyol extracted from the herb *Dracocephalum rupestre* reduced TNF-α, inducible nitric oxide (NO) synthase (iNOS), and glial fibrillary acidic protein (GFAP) expressions, as well as prevented neuronal death, reduced the infarct area, and ameliorated neurological and memory deficits [30]. Ruscogenin isolated from the herb *Ophiopogon japonicas* decreased the infarct size and improved neurological deficits by NF-κB inhibition that suppresses iNOS, COX-2, TNF-α, and IL-1β [31]. The 6-hydroxycleroda-3,13-dien-15,16-olide (PL3) has been extracted from *Polyalthia longifolia* var. *pendula* and was reported to inhibits microglia-mediated inflammation and inflammation-related neuronal cell death by inhibition of the NF-κB signaling pathway [32].

For mental illness, berberine, a major constituent alkaloid isolated from *Coptis chinensis*, prevents depressive-like behaviors in mice by suppressing IL-1β, IL-6, and TNF-α expression levels, as well as microglial activation [33]. Icariin also exerts antidepressant-like effects in rats with mild chronic stress, which may be mediated by an enhanced anti-inflammatory effects by inhibition of NF-κB signaling and the NLRP3-inflammasome/caspase-1/IL-1β axis [34].

### 2.4. Brief Summary of Chinese Herbal Compounds on Neuroinflammation

**Figure 1:**
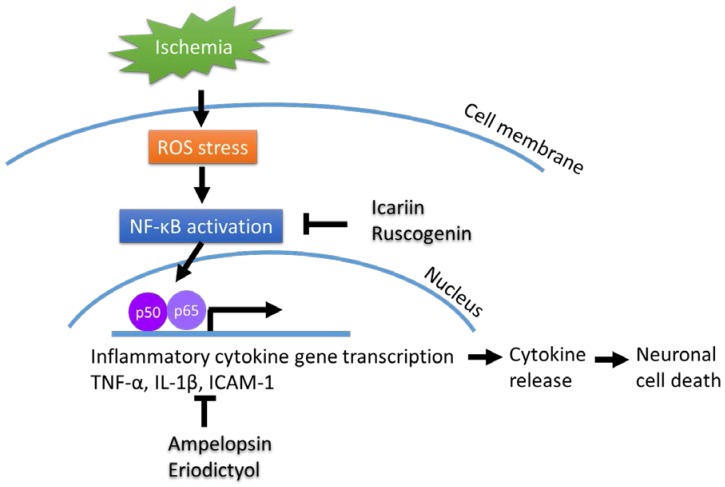
Chinese herbal compound decrease NF-κB activation and cytokines release after ischemia injury.

In summary, Chinese herbs that regulate the NF-κB signaling pathway and its downstream targets, such as IL-1β and TNF-α, usually attenuate inflammatory responses and protect against neuronal death (Figure 1), suggesting that treatment with these herbs may provide potential therapies for neurodegenerative diseases.

## 3. Chinese Herbal Drugs for Neuroprotection via Autophagy

### 3.1. The Importance of Regulating Autophagy in the Nervous System

Autophagy, which has garnered much attention in the past few years, is an intracellular recycling and metabolic process that is critical in different areas of cell physiology. Cytosolic components can be engulfed by the cell membrane and sequestered. After formation of autophagosome, the sequestered organelles and cell debris are delivered to a lysosome; then, the outer membrane of the autophagosome fuses with the lysosome to maintain homeostasis by lysosomal hydrolase [35]. This ensures a dynamic balance between the production and degradation of molecules and cell flow, and any damage to this important intracellular route results in a variety of different diseases such as neurodegenerative diseases [36,37,38], metabolic-related disorders [39], cancer [40], and cardiovascular and pulmonary diseases [41,42].

### 3.2. Autophagy Dysregulation and Neurodegenerative Diseases

It is well-known that many neurodegenerative diseases involve protein aggregation and deposition, such as AD, PD, ALS, and polyQ diseases [43]. Clearance of these abnormal proteins may be the key point to slow down disease progress. Therefore, regulation of autophagy can be a strategy to improve the pathology of neurodegenerative diseases by intracellular cleaning and inhibiting the accumulation of misfolded proteins. Although autophagy plays an important role in neurodegenerative diseases, the detailed mechanism remains unclear.

### 3.3. Chinese Herbal Compounds Promote the Regulation of Autophagy and Improve Neurodegenerative Diseases

Wang et al. summarized autophagy modulators from Chinese herbs for neurodegenerative diseases in the *Journal of Ethnopharmacology* [44]. The study’s authors collected literature from scholarly databases published during a nine-year period (2007–2015) and investigated the effects of Chinese herbal compounds or their natural extracts on regulation of autophagy. We summarize some autophagy regulators from Chinese herbs in terms of the therapeutic effects on neurodegenerative disease, and it can provide new pharmacological applications for drug development.

Overall, a number of articles have been published on modulation of autophagy by Chinese herbal medicine in the past five years. The dual roles of up- or downregulation of autophagy by natural compounds for neuroprotection have been revealed. The impairment of autophagolysosome formation and maturation may contribute to the gradual accumulation of Aβ and phosphorylated tau proteins in AD. It has been reported that resveratrol, luteolin, berberine, *Thamnolia vermicularis* extract, carnosic acid, and ginsenoside compound K derived from different Chinese herbs promote clearance of Aβ accumulations [45,46,47,48,49,50], inhibit the production of hyperphosphorylated tau, and improve the behavioral performance via the adenosine monophosphate-activated protein kinase (AMPK)-dependent, phosphatidylinositol-3-kinase (PI3K)/AKT/mammalian target of rapamycin (mTOR), and AMPK/raptor/mTOR signaling pathways. In contrast, autophagy has been shown to be necessary for apoptotic cell death, placing it upstream of apoptosis, which means that autophagy can induce apoptosis, leading to cell death. In this way, it was reported that triptolide significantly reduces cytotoxicity and apoptosis by inhibiting the autophagic pathway in PC12 cells [51].

Studies have demonstrated that autophagy is also involved in the pathogenesis of PD. Baicalein, hederagenin, carnosic acid, sulforaphane, piperine, triptolide, conophylline, and resveratrol [52,53,54,55,56,57,58,59,60] significantly upregulate autophagy to prevent behavioral deficits, attenuate dopaminergic neuronal loss, neuronal apoptosis, and mitochondrial dysfunction, suppress oligomerized α-synuclein, and restore the interactions of parkin and beclin-1 via the AMPK-mTOR signaling cascade and heme oxygenase-1, LC3II, and p62 signaling. In contrast, oleuropein, baicalein, and cucurbitacin E decreased neuronal death and autophagic flux by inhibiting autophagic initiation in 6-OHDA or MPP^+^-infused substantia nigra (SN) PD models [53,61,62].

In ALS, Berberine upregulated the deregulated mTOR/p70S6K signal and activated an autophagic degradation pathway to promote clearance of TDP-43 [63]. On the other hand, n-butylidenephthalide (Bdph) downregulated autophagy by enhancing p-mTOR, decreasing LC3II to prolong the lifespan, and attenuating motor neuron loss in ALS mice [64,65]. 

The roles of Chinese herbal medicines in other neurodegenerative diseases were published in the past three years. Sulforaphane, neferine, conophylline, and resveratrol eliminated mutant Huntingtin (Htt) aggregates [58,66,67,68], and cleared Htt protein and inclusions against mutant Htt or CAG repeats by activating AMPK-mTOR signaling. Both breviscapine and ginsenoside Rb1 extracted from Chinese herbs played neuroprotective roles by downregulating autophagy. Breviscapine reduced infarct volumes and neuro-functional deficiencies in a cerebral ischemic rat model [69], and ginsenoside Rb1 decreased the loss of motor neurons and promoted functional recovery in a spinal cord injury model [70]. AMP inhibited D-gal-induced apoptosis and rescued impaired autophagy of neurons by upregulating SIRT1 and downregulating the mTOR signal pathways [71].

### 3.4. Brief Summary of Chinese Herbal Compounds Involved in Regulating Autophagy

**Figure 2:**
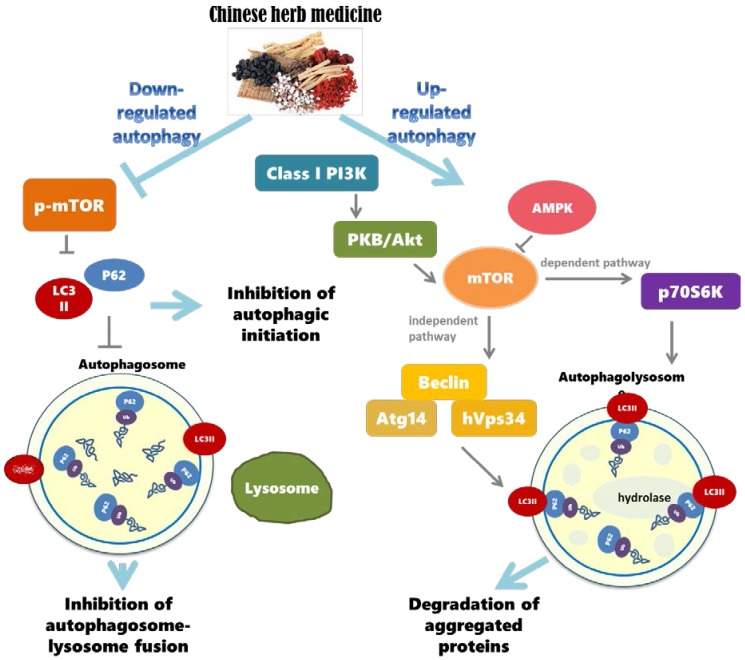
Autophagy regulation by Chinese herb medicine.

Many studies have investigated the regulation of autophagy by extracts of Chinese herbal medicines. Results suggest that herbal compounds derived from Chinese herbal medicines are able to protect the functions of neurons against mutant proteins and protein aggregates. These extracts may regulate autophagy via various pathways, especially the PI3K/AKT/mTOR pathway. The mTOR is an important key for autophagy, and most studies reported that those extracts from Chinese herbal medicines may regulate autophagy via mTOR signaling (Figure 2). Results indicated that herbal compounds extracted from plants hold great potential for treating neurodegenerative diseases, and targeting the modulation of autophagy for therapeutic effects is a great strategy for neuroprotection.

## 4. Chinese Herbal Drugs for Neuroprotection via Reduced ER Stress

### 4.1. ER Function and Maintenance of Neurons

The ER is a multifunctional organelle, involved in lipid and sterol synthesis, protein post-translational modifications, and protein transport [72]. The neuronal ER also mediates the Ca^2+^ intracellular signal, producing local or global cytosolic calcium in neurons [73]. Neurons are highly bipolar cells with very long dendrites and axons. The shape of the ER in neurons is between those of dendrites and axons. ER tubules extend along microtubules to build up the transport network [74]. The neuronal ER network is dynamically remodeled during neuronal development. During dendrite and axon developmental processes, the ER rapidly extends bidirectionally and retains protein trafficking from the soma [75]. The neuronal ER in soma and dendrites has an abundant ribosomal distribution [76]. The dendritic ER structure is modulated by external cellular signals, when type I metabotropic glutamate receptors are activated [77], and the axonal ER is rich in Ca^2+^ ATPase and IP_3_R [78]. Thus, the ER is a very important organelle during neuron development.

### 4.2. The Role of ER Stress in Neuronal Diseases

**Figure 3:**
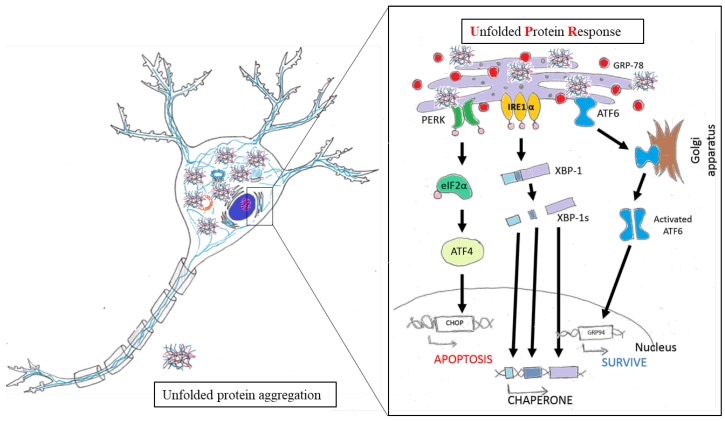
Schematic representation of the unfolded protein response pathway.

Most neurodegenerative diseases are caused by misfolded protein accumulation. Proteins that accumulate abnormally in the ER generate stressful conditions termed ER stress. When a cell faces this situation, it activates the unfolded protein response (UPR) [79]. In the initial stage, the UPR is regulated by three ER stress sensors: inositol-requiring protein-1 (IRE1), activating transcription factor-6 (ATF6), and protein kinase RNA (PKR)-like ER kinase (PERK). When the UPR is activated, neurons downregulate protein translation, enhance translation of chaperone protein, and begin to produce chaperones and other components for the accurate folding of proteins [80,81]. However, when facing prolonged ER stress, the cell activates the UPR-apoptosis process. C/EBP homologous protein (CHOP) and caspase-12 are induced by ATF4 and X box-binding protein-1 (XBP-1) [82]. In AD, overexpressed Aβ forms oligomers, which accumulate in the ER lumen and activate the proapoptotic ER stress response. Brains of AD show high expressions of UPR markers, such as GRP78 and p-PERK, in the cortex and hippocampal region [83,84,85,86]. Similarly, α-synuclein accumulates in the ER then disrupts protein transport from ER to Golgi in PD [87,88]. In ALS, mutant SOD1 was observed in the ER and was found to be associated with motor neuron death [89,90,91,92]. According to those studies, UPR signaling plays an important role in neuroprotection (Figure 3). Thus, discovering new compounds to reduce ER stress and restore ER function is very important.

### 4.3. Chinese Herbal Drugs Reduce ER Stress and Benefit Nervous System

Astragaloside IV has been extracted from the roots of *Astragalus membranaceus*, and was found to inhibit the ER stress apoptosis pathway [93]. Astragaloside IV also promoted neurite outgrowth and increased survival of dopaminergic neurons in a PD model [94]. Baicalein, which is isolated from roots of *Scutellaria baicalensis* and *S. lateriflora*, were previously introduced in the autophagy section, also have potential to apply for the treatment of PD via ER stress regulation. It inhibits CHOP expression in the HT-22 cell line and rotenone-induced apoptosis in dopaminergic SH-SY5Y cells by inhibiting brefeldin A (BFA)-induced CHOP, GRP78, ATF6, and p-eIFα expressions, and reducing apoptotic caspase-3 and caspase-12 splicing [95,96]. Crocin, isolated from saffron, has been used in TCM for decades. Crocin attenuates MPP(+)-induced cell apoptosis by inhibiting CHOP expression and the Wnt signaling pathway in PC12 PD cell model [97]. Crocin also inhibited the effect of tau protein aggregation in AD in an in vitro model [98]. Interestingly, heroin-addicted rats showed significant downregulation of PERK, eIF2a, and CHOP expressions and a reduction in neuron apoptosis [99]. Taken together, ER stress in neural diseases is well connected with herbal compound treatment.

### 4.4. Brief Summary of Chinese Herbal Drugs Involved in ER Stress

Unfolded protein response pathway is highly involved in the first step of neurodegenerative diseases, especially in amyloid triggered diseases including AD, PD, ALS, and Huntington disease (HD). According to these evidences, there are two candidate pathways for treating neurodegenerative diseases. One is to inhibit the ER stress-activated apoptotic pathway, and the other one is to enhance chaperone protein translation. However, there are still few Chinese herbs derived pure compounds were identified to improve ER function in neurodegenerative models. Only astragaloside IV, baicalein, and crocin were demonstrated to reduce ER stress or ER-stress-induced apoptosis directly and benefit neuron survival. Therefore, there is still great potential to study the herb-derived compound on ER stress regulation.

## 5. Chinese Herbal Drugs for Neuroprotection via Glucose Metabolism

### 5.1. Effects of Glucose Metabolism on Neurophysiology

The energy requirements of the brain are relatively higher than other parts of the body, particularly in humans. About 20% of the body’s energy consumption occurs in less than 2% of a person’s weight [100]. The brain has specialized glial cells, astrocytes, which are responsible for the delivery, production, storage of brain energy, and metabolism of neurons [101]. That is, neurons rely on glia cells and astrocytes to supply their extreme energy costs and maintain functions. The glucose metabolism pathway and the glucose level in the brain have potential to alter neuron function and survival. Moreover, glucose metabolism is also interconnected with the production of major neurotransmitters as discussed below. Maintaining the balances of glucose uptake maybe one key factor to keep brain neurons healthy.

### 5.2. Dysregulation of Glucose Metabolism in Neurodegenerative Diseases

The correlation of diabetes (one major glucose metabolism disease) and neurodegenerative disease, especially dementia and AD, were reported recently. People with diabetes or metabolism diseases have higher risk of neurodegenerative diseases [102,103]. To emphasize the importance of metabolism dysregulation on neurodegenerative diseases, AD was also suggested as “Type 3 diabetes” [104]. Looking into the mechanisms, the dysregulation of systemic glucose metabolism, such as insulin deficiency or insulin resistance, may also influence the uptake of glucose in human brain. Many studies showed a reduction in insulin production and glucose uptake in the aged, dementia brains [105], and even in patients with diabetes [106]. Dysregulation of glucose metabolism can lead to reduce the energy source, increase oxidative stress and inflammatory response, then damage the brain neurons in AD [107]. In addition, use of fluorodeoxyglucose uptake by PET scans exhibited a reduction of glucose in Aβ damaged brains [108]. These results reveal correlations between glucose metabolism and neurodegenerative diseases.

### 5.3. Potential of Chinese Herbal Drugs to Improve Neural Metabolism

A triterpenoid compound*,* maslinic acid (2-α, 3-β-dihydroxyolean-12-en-28-oic acid), isolated from the wax skin of *Olea europaea*, can increase glycogen synthesis and prevent norepinephrine-induced glycogenolysis in cortical astrocytes [109]. Qian et al. demonstrated that maslinic acid ameliorates neuron injury and apoptosis which accompanied the inhibition of oxygen-glucose deprivation-induced NO production and iNOS expression [110]. The effect of baicalein on preventing neuronal death induced by glutamate and glucose deprivation was also examined [111]. These compounds may have potential for neuronal diseases treatment. Resveratrol has been studied for decades. Its neuroprotective mechanism varies, such as its effects not only on oxidative damage and chronic inflammation, but especially on activating expression of sirtuins, a protein involved in glucose metabolism [112]. Supplementation with resveratrol for 26 weeks (at 200 mg/day) enhanced memory performance in healthy older adults [113]. Moreover, the above double-blind study showed that resveratrol intake significantly reduced serum glycated hemoglobin (HbA1c) compared to a placebo group, and the decreased HbA1c was significantly correlated with an individual’s functional connectivity. There indeed is an effect of glucose metabolism on neural function. This is a great potential to benefit neurodegenerative diseases via metabolism regulation with Chinese herb drugs. Although there are still few herb drugs targeting on systemic and nerve system metabolism, a more-detailed assessment is forthcoming to clarify this issue.

## 6. Chinese Herbal Drugs for Neuroprotection via Neurotransmitters and Synaptic Function

### 6.1. Neurotransmitters on Synapse and Neural Function

Synapse is the basic component for neural communication and neural information flow in nervous system. Stimulations of neurons benefit the maintenance of neuronal function and survival. Neurotransmitters play the major role on synaptic function. The life cycle of neurotransmitter including the production, packaging, release, recycling, and postsynaptic uptake [114]. Glutamate, acetylcholine (ACh), and γ-aminobutyric acid (GABA) are common neurotransmitters in CNS. Glutamate and ACh are excitatory neurotransmitters [115], and GABA is inhibitory neurotransmitter [116]. The tricarboxylic acid (TCA) cycle is further extended to produce glutamate and GABA in the CNS, called “the GABA shunt” [117]. Pyruvate is oxidized to acetyl-CoA in mitochondria, and acetyl-CoA and choline are the material of ACh that synthesized by choline acetyltransferase [118]. Released neurotransmitters can be recycled or re-uptake by nearby astrocytes or pre-/post-synaptic terminals. The released glutamates from presynaptic neurons were recycled by astrocytes via the glutamate–glutamine cycle. Glutamate is converted into glutamine by glutamine synthetase in astrocytes then shuttled to neurons [101]. Released GABA was uptake by GABA transporter in presynaptic terminal. In the synapse cleft, released ACh is broken down into choline and acetate by acetylcholinesterase, and choline is transported into the axon terminal as the recycled material of ACh. Overall, the pre-synapse axons, post-synapse dendrites, and astrocytes can re-uptake released neurotransmitter with re-uptake pump to recycle the neurotransmitter around the synapse as materials to synthesis new neurotransmitter.

### 6.2. Neurotransmitters and Synaptic Function in Neurodegenerative Diseases

Many reports demonstrated the misfolded amyloids would accumulate around the synapses and block the releasing and recycling of neurotransmitter [119,120,121]. The dysregulation of neurotransmitter also causes the abnormality of post-synapse stimulation, including hyper-activation and hypo-activation, and causes neuron disfunction or death [122,123]. It was revealed decades ago that inhibiting NMDA receptors prolongs survival in patients with ALS [124]. A previous study also showed that Aβ is correlated with extracellular GABA levels [125], which raised the theory of the involvement of GABA in the physiopathology of AD [126]. Pimlott et al. demonstrated the reduction of nicotinic ACh receptor (nAchR) in AD, PD, and dementia with Lewy bodies [127].

### 6.3. Chinese Herbal Drugs Improve Neurotransmitter Production and Synaptic Function

Our recent data revealed that a small molecule from the root of *Angelica sinensis* has therapeutic potential in SCA3. This small molecule, Bdph, regulates tryptophan metabolism and decreases the downstream neurotoxic product, quinolinic acid (QA, a NMDA receptor agonist) [128]. Moreover, Bdph reduces Aβ40 secretion and attenuates AD-like cytopathy in AD models [129]. Huperzine A has been extracted from the firmoss *Huperzia serrata* and competes with the NMDA receptor on polyamine-binding sites [130]. Huperzine A has treatment potential for AD and vascular dementia [131]. The type A GABA receptor is a postulated target in age- and AD-related neuronal degeneration [125]. The neuroprotective effect of kava, a modulator of the GABA receptor, has been reviewed for the detailed mechanism of its antianxiety and sleep-inducing abilities [132]. Some other herb extracts were reported to improve neurotransmitter and synapse function. However, the major functional compounds are still unclear and need further identification [133]. Moreover, the Chinese herbal drugs that benefit neurons mentioned in the above sections may also have potential to work on synaptic plasticity. Further studies of herbal drugs on synapse and neurotransmitter would provide new direction of herbal drugs in neurodegenerative diseases.

## 7. Discussion

The evidence suggests that Chinese herbal drugs have neuroprotective abilities in various neurodegenerative disease models in different ways (Table 1). One is to attenuate the inflammatory response through cytokine regulation, and several previous experiments showed that the IL-1β and TNF-α signaling pathways could be inhibited to benefit neurons in neurodegenerative diseases. Regulating the mTOR signaling pathway in autophagy is also a neuroprotective strategy, and herbal compounds from Chinese herbs have different effects on this signaling pathway. Due to opposite roles of the mTOR pathway in neurons being still largely unknown, the detailed mechanisms of autophagy on neurodegenerative diseases are still in need of further study. Several neurodegenerative diseases have unusual protein misfolding and accumulation in the ER, and herbal compounds inhibit UPR-related apoptosis, which prevents neurons undergoing ER stress from dying. Due to the correlation between metabolic abnormalities and neurodegenerative diseases, such as AD, revealed recently, there is a large potential to apply herb compounds to maintain metabolism balance to combat neurodegenerative diseases. Besides the glucose metabolism, there are also some mechanisms and metabolic pathways related to neuroprotection not mentioned above. As neurons can die due to aberrant protein accumulation, the efficiency of waste clearance and improving brain blood flow are important. Furthermore, nutrient support from glial cells to neurons is also important. Although herbal compounds have neuroprotective abilities, the off-target effect of herbal compounds remains a concern. Some of the herbal compounds involved more than one mechanism, such as AMP, baicalein, berberine, Bdph, and resveratrol, and the neuroprotective specificity by these compounds still need to be confirmed. Moreover, herbal compounds show opposite impact on the mTOR pathway for neuroprotection in neurodegenerative disease models. This indicates that autophagy is complex in neurodegenerative diseases and the regulation of autophagy for neuroprotection may vary with the disease type and stage. Inhibiting UPR-related apoptosis prevents neurons undergoing ER stress from dying, but the ER stress is still high in neurons. The way to inhibit ER-stress-induced cell death may be insufficient to cure neurodegenerative diseases, and releasing ER stress may be needed. As the progress of neurodegenerative diseases is complicated and the herbal compounds have different impacts on the nervous system, combinations of compounds may be needed. In addition, therapeutic strategies may vary with the type and progression of disease.

## Figures and Tables

**Table 1:** Chinese herbal compounds have neuroprotection ability to various neurological disorders through different mechanisms and metabolisms.

Herbal Drugs	Plant Sources	Structure	Involved Mechanism (s)	Treated Model	Main Citations
6-hydroxycleroda-3,13-dien-15,16-olide (PL3)	*Polyalthia longifolia var. pendula*	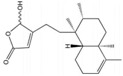	cytokine regulation	LPS-induced model	[32]
Ampelopsin	*Ampelopsis grossedentata*	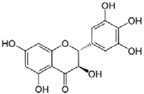	cytokine regulation, autophagy regulation	Stroke, brain aging	[29,71]
Astragaloside IV	*Astragalus membranaceus*	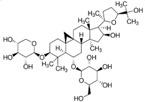	ER stress	PD	[94]
Baicalein	*Scutellaria baicalensis and S. lateriflora*	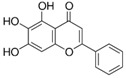	autophagy regulation, ER stress	PD	[53,54,96]
Berberine	*Coptis chinensis*	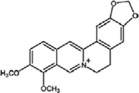	cytokine regulation, autophagy regulation	depression, AD, and ALS	[33,47,63]
Breviscapine	*Erigerin breviscapus*	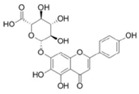	autophagy regulation	cerebral ischemic	[69]
n-butylidenephthalide	*Angelica sinensis*	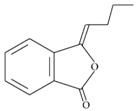	autophagy regulation, neurotransmitters and synaptic function	ALS, SCA3, and down syndrome model	[64,65,128,129]
Carnosic acid	*Rosmarinus officinalis*	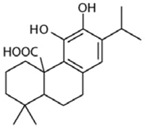	autophagy regulation	AD and PD	[50,55]
Conophylline	*Ervatamia microphylla*	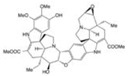	autophagy regulation	PD and HD	[58]
Crocin	*Crocus sativus*	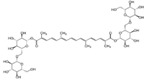	ER stress	PD and AD	[97,98]
Cucurbitacin E	*Wilbrandia ebracteata*	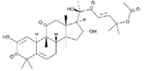	autophagy regulation	PD	[62]
Eriodictyol	*Dracocephalum rupestre*	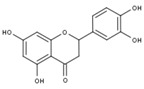	cytokine regulation	stroke	[30]
Ginsenoside compound K	*Panax ginseng*	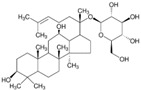	autophagy regulation	AD	[46]
Ginsenoside Rb1	*Panax ginseng*	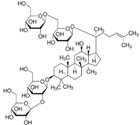	autophagy regulation	spinal cord injury	[70]
Hederagenin	*Hedera helix*	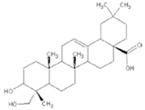	autophagy regulation	PD	[59]
Huperzine A	*Huperzia serrata*	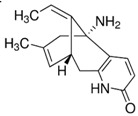	neurotransmitters and synaptic function	AD	[130,131]
Icariin	*Epimedium segittatum*	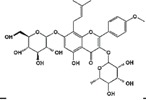	cytokine regulation	AD, stroke, depression	[25,28,34]
Icariside II	*Epimedium brevicornum*	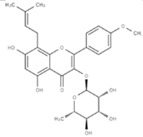	cytokine regulation	AD	[26]
Isobavachalcone	*Psoralea corylifolia*	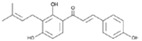	cytokine regulation	PD	[24]
Luteolin	*Lonicera japonica*	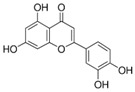	autophagy regulation	AD	[48]
Maslinic acid	*Olea europaea*	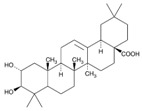	glucose metabolism	oxygen-glucose deprivation-induced injury model	[110]
Neferine	*Nelumbo nucifera*	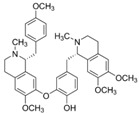	autophagy regulation	HD	[68]
Oleuropein	*Olea europaea*	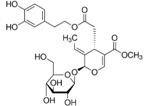	autophagy regulation	PD	[61]
Paeonol	*Cortex Moutan*	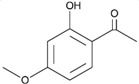	cytokine regulation	PD	[23]
Piperine	*Piper longum L.*	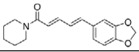	autophagy regulation	PD	[57]
Resveratrol	red wine	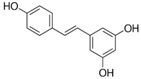	autophagy regulation, glucose metabolism	AD, PD, HD, and healthy older adults	[45,56,67,113]
Ruscogenin	*Ophiopogon japonicas*	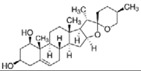	cytokine regulation	Ischemic stroke	[31]
Sulforaphane	*cruciferous vegetables*	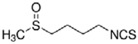	autophagy regulation	PD, HD	[60,66]
Tetrandrine	*Stephania tetrandra*	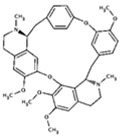	cytokine regulation	AD	[27]
Thamnolia vermicularis extract	*Thamnolia vermicularis*	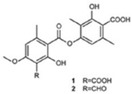	autophagy regulation	AD	[49]
Triptolide	*Tripterygium wilfordii*	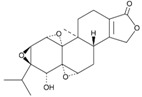	autophagy regulation	AD, PD	[51,52]

ER, endoplasmic reticulum; LPS, lipopolysaccharide; PD, Parkinson’s disease; AD, Alzheimer’s disease; ALS, amyotrophic lateral sclerosis; SCA3, spinocerebellar ataxia type 3; HD, Huntington disease.
